# A synchrotron X-ray diffraction deconvolution method for the measurement of residual stress in thermal barrier coatings as a function of depth

**DOI:** 10.1107/S1600576716013935

**Published:** 2016-10-21

**Authors:** C. Li, S. D. M. Jacques, Y. Chen, D. Daisenberger, P. Xiao, N. Markocsan, P. Nylen, R. J. Cernik

**Affiliations:** a School of Materials, University of Manchester, Manchester M13 9PL, UK; b Diamond Light Source, Chilton, Didcot, Oxfordshire OX11 0DE, UK; c Production Technology Centre, University West, Trollhättan, Sweden

**Keywords:** thermal barrier coatings, synchrotron glancing-angle X-ray diffraction, stress mapping, flat plate powder diffraction

## Abstract

The average residual stress distribution as a function of depth in an air plasma-sprayed yttria stabilized zirconia top coat used in thermal barrier coating (TBC) systems was measured using synchrotron radiation X-ray diffraction in reflection geometry on station I15 at Diamond Light Source, UK, employing a series of incidence angles. The stress values were calculated from data deconvoluted from diffraction patterns collected at increasing depths. The stress was found to be compressive through the thickness of the TBC and a fluctuation in the trend of the stress profile was indicated in some samples. Typically this fluctuation was observed to increase from the surface to the middle of the coating, decrease a little and then increase again towards the interface. The stress at the interface region was observed to be around 300 MPa, which agrees well with the reported values. The trend of the observed residual stress was found to be related to the crack distribution in the samples, in particular a large crack propagating from the middle of the coating. The method shows promise for the development of a nondestructive test for as-manufactured samples.

## Abstract 2

The strain profile in a YZrO_3_ thermal barrier coating has been measured and shows possible mechanisms of in-service failure. The method relies on deconvoluting diffraction patterns at different depths for the reflection geometry used. The nondestructive test could be applied to whole fabricated components

## 1. Introduction   

The residual stress generated during the manufacture and use of an industrial component is a commonly occurring problem (Genzel *et al.*, 2011 ▸), especially for layered structures such as coatings (Li *et al.*, 2016 ▸) and welded or brazed joints (Cao *et al.*, 2015 ▸). These residual stresses can eventually cause catastrophic failures such as spallation or cracking (Ohtsuka *et al.*, 2007 ▸). Since these residual stresses are closely related to the failure and lifetime of the components, it is very important to measure the residual stress distribution in these structures. This is particularly true for fast deposition methods to cover large component areas such as air plasma-sprayed (APS) thermal barrier coatings (TBCs).

TBCs are usually made from yttria stabilized zirconia (YSZ) with 8 wt% yttria applied on the surface of turbine blades that are working at very high temperature (Chen *et al.*, 2015 ▸, 2012 ▸; Garces *et al.*, 2014 ▸). The coating system consists of three main parts: a top coat, a bond coat used to join the top coat and substrate, and a substrate which is usually made of a nickel superalloy. During use a layer of thermally grown oxide (TGO, predominately α-alumina) forms on the bond coat surface, which inhibits further oxidation. The ceramic coating enables the engines to be operated at higher temperatures, to prevent the alloy melting and to improve its operational efficiency. However, the failure mechanism of TBCs has not been fully understood (Clarke & Levi, 2003 ▸; Evans *et al.*, 2001 ▸), and reliable nondestructive methods to predict the likely lifetime of TBCs need to be developed. For APS TBCs, the failure usually happens in the top coat near the interface region (Rabiei & Evans, 2000 ▸; Trunova *et al.*, 2008 ▸; Beck *et al.*, 2008 ▸). The driving force for the generation and propagation of cracks is believed to be the residual stress in the coating, originating from the mismatch of the thermal expansion coefficient (CTE) between the top coat and the substrate. Determining the stress distribution in the top coat can help to predict the site of failure in the coating. Thus it is very important to investigate the stress distribution as a function of depth in TBCs to help give a better understanding of the failure mechanism of these coatings.

The residual stress is known to be closely related to the sample microstructure, and the microstructure of an APS TBC is very complex (Evans *et al.*, 2001 ▸). This microstructure includes pancake-like features that form on deposition which are known as ‘splats’. Further to these features the TBC typically develops inter-splat cracks and a rumpled interface between the top coat and the bond coat. Many models are unable to incorporate all these features, which makes then less reliable compared with the modelling of simpler, more homogeneous, structures. Measuring the residual stress directly can be considered a more reliable approach to obtain a stress/strain profile, and there are a number of ways to measure residual stresses in TBCs. The curvature method (Clyne & Gill, 1996 ▸; Godoy *et al.*, 2002 ▸) is a commonly used and convenient way to estimate residual stresses; however this method is destructive and only gives an average value of the residual stress through the coating instead of giving the depth-resolved stress profile. Raman spectroscopy (Mao *et al.*, 2010 ▸; Liu *et al.*, 2012 ▸) and indentation methods (Zhao & Xiao, 2006 ▸; Zhu *et al.*, 2012 ▸) have also been applied to measure the residual stress in TBCs. For these two methods, to achieve the stress distribution as a function of depth, the samples need to be cross sectioned, ground and polished, which inevitably changes the strain distribution inside the sample. Also, for the Raman method, since zirconia is transparent for most lasers, the beam will spread inside the coating (Liu *et al.*, 2013 ▸). This leads to considerable uncertainty in determining the measured sample volume, which in return affects the resolution of the measurement. For the indentation method, the penetration depth of most indenters is limited (∼5 µm) so the measured stress is mostly from the surface. Laboratory-based X-ray diffraction is a commonly used method to measure residual stress in samples (Chen *et al.*, 2010 ▸). However, owing to the high absorption in zirconia, the penetration depth (∼10 µm for standard laboratory-based X-rays) is usually too small to measure the stress in the interface region, which is arguably of the greatest importance regarding the failure mechanism of TBCs.

To obtain the stress distribution with depth by X-ray diffraction (XRD), the X-ray source used must be able to offer high energy (to achieve reasonable penetration depth), high flux, and a parallel and monochromatic beam. Synchrotron sources can fulfil these requirements. Some studies (Thornton *et al.*, 2005 ▸, 1999 ▸) have utilized synchrotron sources to measure the residual strain distribution in TBCs to obtain the strain distribution as a function of depth. However, the authors heat annealed the samples and cut them, which is known to cause relaxation and redistribution of the stress. In addition, the sample length was only 3.5 mm, which is too small to represent the stress state in actuality. There are reports of similar methods (Weyant *et al.*, 2010 ▸) to obtain the residual stress distribution as a function of depth in TBCs where an increasing trend of compressive stress from the surface to the interface was found. However, the sample size that the authors used was again too small (2.5 mm in length) to represent a realistic operational situation. A recent study (Knipe *et al.*, 2014 ▸) used transmission geometry to investigate the strain distribution and response of a TBC to mechanical and heat loading. Here the sample was curved, so at deeper penetration depths, the average depth strain was measured. Although the experiment in transmission geometry is able to show a trend with depth, this geometry is limited by the penetration depth of the X-rays. Reflection geometry can avoid this problem and it can be used to measure the strain/stress nondestructively on any size or shape of sample, so making the approach attractive for portable industrial inspection systems. In general, the thickness of the coating is much smaller than the other dimensions of the sample/component and thus reflection geometry requires a smaller X-ray beam path inside the sample than transmission geometry. The main disadvantage of reflection geometry is the need to deconvolute the XRD data from various depths or layers in the TBC after collection; the method is discussed in detail in the following section.

To date, two main methods to achieve a profile as a function of depth have been published. The first is an energy-dispersive method (Meixner, Fuss, Klaus & Genzel, 2015 ▸; Meixner, Fuss, Klaus, Genzel & Genzel, 2015 ▸) which is an extension of the strain scanning methods that define a gauge volume in the sample. The second involves using a variable incident angle to obtain the desired penetration depth. However, only the average value of the stress from the surface to the maximum penetration depth can be obtained (Mittemeijer & Welzel, 2013 ▸). Some research has been carried out to deconvolute the diffraction information of a material at each depth from the average value using a Laplace transformation or numerical methods (Mittemeijer & Welzel, 2013 ▸; Stefenelli *et al.*, 2013 ▸). These methods can be used to deconvolute the stress information at different depths. Currently, no measurement of residual stress distribution as a function of depth in TBCs in reflection geometry has been reported.

This paper introduces a method to extract the residual stress as a function of depth in thick coatings (TBCs) using high-energy monochromatic synchrotron X-rays and an area detector. A method to deconvolute the biaxial stress value at each successive depth inside the coating has been developed and is described herein.

## 2. Deconvolution method   

**Figure 1:**
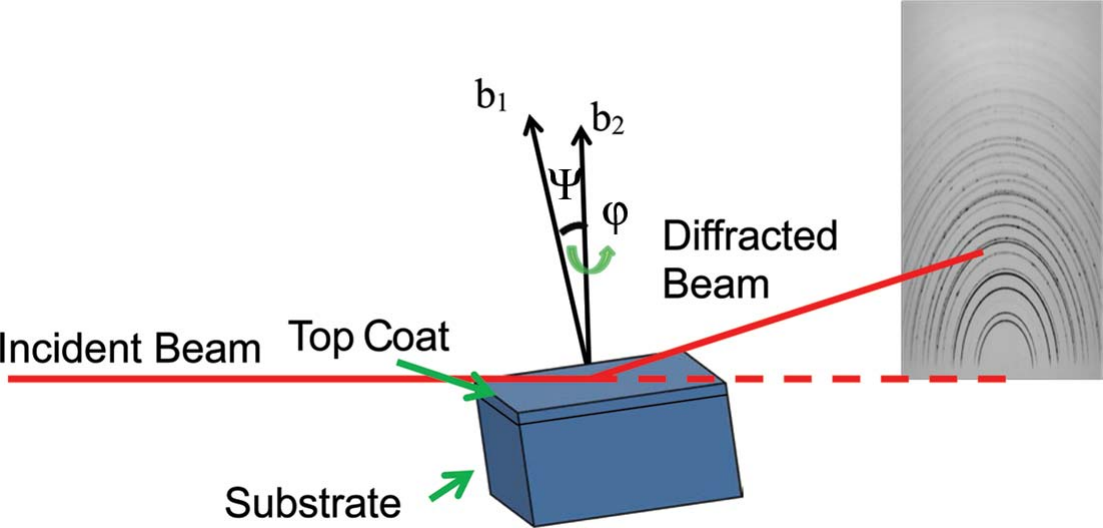
A schematic diagram of the experimental geometry, showing the path of the beam and portions of the Debye–Scherrer rings collected on the detector. These rings were partially integrated in segments to form one-dimensional diffraction patterns to be analysed by Rietveld refinement. The DS rings can be integrated at different azimuthal angles to give in-plane and out-of-plane information.

To analyse the stress distribution inside the coating, the 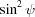 method was initially utilized to calculate the in-plane stress. This method (Noyan & Cohen, 2013 ▸) is based on the peak shift of the XRD pattern caused by the residual stress. During the calculation, one reflection (diffraction plane) is chosen and the stress in different directions can be calculated by
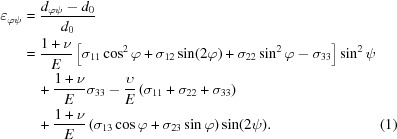
where 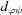 is the *d* spacing measured at φ and ω, *d*_0_ is the stress-free *d* spacing, ν is Poisson’s ratio, *E* is Young’s modulus, and σ is the stress. As shown in Fig. 1 ▸, **b**_1_ is the angle bisector between the incident and the diffracted beams, and **b**_2_ is the normal of the sample surface. The angle 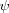 is the angle between **b**_1_ and **b**_2_, and the angle 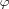 denotes the rotation of the specimen around the specimen surface normal **b**_2_.

The residual stress in the TBC is generated by the mismatch between the CTE of the top coat and the substrate. Thus an in-plane stress state is expected in the coating after cooling, which means 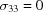 in equation (1). In the measurement, the 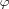 angle was not changed and remains zero. Then the equation can be simplified to
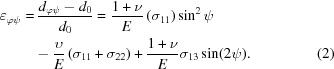
It can be seen from equation (2) that if the shear stress 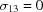 the slope of 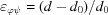 plotted against 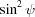 is proportional to the in-plane stress 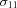. The in-plane compressive stress is considered to be closely related to the failure mechanism of the TBC. The *d* spacing measured from the direction perpendicular to the surface is usually taken as the strain-free *d* spacing. Because of the Poisson effect, taking the *d* spacing measured from the direction perpendicular to the surface as the strain-free *d* spacing is not accurate. But usually for the 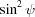 method, this error can be neglected. In this paper, two diffraction planes, (024) and (312), were selected to calculate the in-plane stress in the coating. These two peaks were chosen because they are at a relatively high angle in our pattern and are hence more stress sensitive. The overlap of the 015, 033 and 321 peaks at higher 2θ angles makes peak deconvolution more difficult with a negative effect on the stress calculation accuracy. The elastic constants (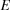 and 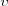) of the two planes were obtained from the *Isodec* software (Gnäupel-Herold, 2012 ▸), and the quantity 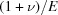 was set as 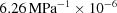 for the (024) diffraction plane and 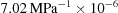 for the (312) diffraction plane. In this way the average residual stress distribution as a function of depth was estimated.

To help understand the failure mechanism of TBCs, understanding the precise residual stress at a given depth is important and useful. To obtain the precise residual stress distribution at a certain depth, the following procedures were carried out. The average residual stress measured by the X-rays from different penetrated layers can be represented (Kumar *et al.*, 2006 ▸) by
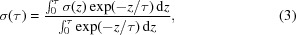
where 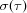 is the average stress (the stress measured by the 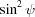 method) in the depth range from the surface to 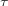 below the surface, and 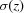 is the residual stress in a thin sub-layer (defined by our method) at depth *z* which needs to be solved. For each incident angle, the corresponding penetration depth will give a value for 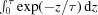. Thus, the focus is on the solution of 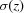 from
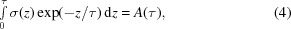
where 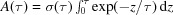. This equation can be regarded as one form of the Fredholm integral equation (Wu *et al.*, 2002 ▸; Broadhurst *et al.*, 2005 ▸) of the first kind:
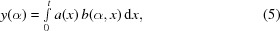
where 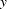
(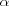) is the direct measured value, 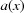 is the solution needing to be determined and 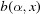 is an absorption term as a function of incident angle 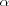 and depth *x*. The equation can be solved for each 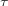 value to get the residual stress distribution 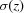. However the Fredholm integral equation of the first kind is ill conditioned (Broadhurst *et al.*, 2005 ▸). So a very small error in *A* can result in very large errors in 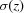. Thus the determination of an accurate value of 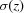 is a challenge. This kind of problem is also encountered in deconvoluting the XRD pattern as a function of depth (Broadhurst *et al.*, 2005 ▸). The most commonly used way to solve this problem is by linear squares regulation to eliminate large errors. We have used a similar method with the integration being solved in a numerical way. The coating can be divided into *n* sub-layers and the residual stress value in each sub-layer can be considered to be homogeneous. Thus the value of 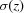 can be found at discrete values 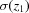, 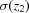,…, 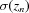 at different depths. The problem can be represented by the matrix equation
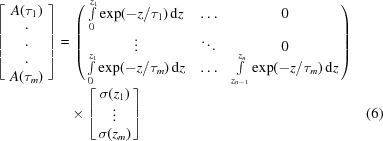
or, for simplicity, written as 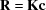. In the general case 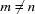 and thus the equation may not always be analytically solvable. However, an iterative approach using the least squares method is a good way to approach this problem. The aim is to obtain the minimum value of 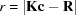 or 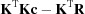. Since this kind of equation is ill conditioned, a regularization technique was used (Broadhurst *et al.*, 2005 ▸). An extra term was added to the equation, 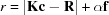, where 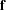 is a function of the solution chosen to regularize the system (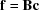) and 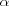 is a weighting parameter. The first order of regularization was used to obtain the solution in this paper:
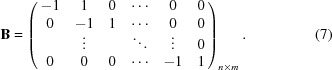
Thus, the solutions are the **c** values minimizing the formula
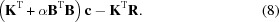


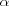
 was set as 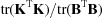.

## 3. Experimental details   

Our TBC samples were fabricated at University West by air plasma spraying. They consist of a YSZ top coat (∼250 µm thick) and an NiCoCrAlY bond coat (∼150 µm thick) deposited on a Hastelloy X superalloy substrate. Before heat treatment, the samples were carefully cut into 10 × 10 × 5.4 mm pieces using a slow-speed SiC abrasive cutting wheel in a precision cutoff machine. The samples were then heat treated in a Carbolite muffle furnace at 1423 K for 100 h.

The XRD measurements were carried out on beamline I15 at Diamond Light Source, UK, in reflection geometry. This is shown schematically in Fig. 1 ▸; a PerkinElmer flat panel detector was used to collect the full Debye–Scherrer diffraction rings (at least those visible above the sample). The detector consisted of 2048 × 2048 pixels and each pixel had dimensions of 200 × 200 µm. The detector was mounted orthogonally to the beam and the beam centre was aligned toward the lower middle of the detector. The detector was offset upward to collect as many as possible of the Debye–Scherrer rings from the reflection geometry. The lower part of the diffraction rings was occluded by the sample. The working distance of the detector was 900 mm, calibrated by *FIT2D* (Hammersley, 2016 ▸) using a CeO_2_ NIST standard. The energy of the beam was set at 72 keV and the beam size was adjusted to 20 × 20 µm. At the beginning of the measurement the sample surface was aligned parallel to the beam and the height of the sample was adjusted so that the beam has grazing incidence on the surface. The sample was then tilted to increase the incident angle to increase the penetration depth. The incident angle was increased from 0 to 3.8° with a step size of 0.38°. The incident position of the X-rays was also adjusted accordingly to make the centre the footprint of the X-ray beam fall in the middle of the coating. When the incident angle rose to 3.8°, peaks from the nickel-based bond coat were observed, indicating that the beam had reached the top coat/bond coat interface. After the measurements, the Debye–Scherrer rings were partially integrated in segments (cake slices) by *FIT2D* in five sections from 40 to 60°, 60 to 80°, 80 to 100°, 100 to 120° and 120 to 140°, respectively. The five sections represent the ψ angles (the angle between the normal of the sample surface and the normal of the equipment system) of 40, 20, 0, −20 and −40°. The caked rings were then integrated into one-dimensional diffraction patterns.

Since the measurements were carried out in near-grazing-incidence geometry, the path length of the incident and reflected X-ray beam is not symmetric inside the sample. Thus before further analysis, the absorption correction (Ryding *et al.*, 2012 ▸) for the pattern was carried out. The equation 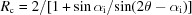 was applied, where 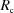 is the intensity ratio between the calibrated pattern and the uncalibrated pattern, 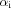 is the incident angle of the beam, and 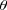 is the Bragg angle.

At very low incident angles refraction can become significant. In our measurements, some incident angles are relatively low (0.38 and 0.76°), and thus the effect of refraction needed to be calculated. According to work carried out by Lim *et al.* (1987 ▸), the index of the X-rays is slightly less than unity and can be calculated by
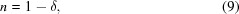


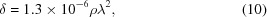
where 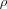 is the density of the material probed by the X-rays and 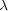 is the wavelength of the X-rays, which is 0.17222 Å in our experiment. The theoretical density of fully dense tetragonal zirconia is 6.2 g cm^−3^. The δ term in equation (9) for zirconia was 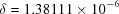. The shift of 2θ can be calculated by
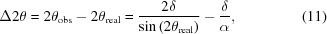
where 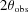 is the measured 2θ value without refraction correction, 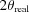 is the 2θ value after refraction correction and 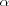 is the incident angle in radians. Typically scans with an incident angle less than 1° need to be calibrated because of the refraction effect. In this experiment, the scans with incident angles of 0.38 and 0.76° were considered, but the peak shift caused by refraction is almost independent of the 2θ angle (Lim *et al.*, 1987 ▸) so the 2θ value was taken as 8.2°, which is the average of the range refined (6.2–10.2°). The calculated 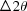 is −0.0011° for the incident angle of 0.38° and −0.00047° for the incident angle of 0.76°. This calculation was based on a fully dense material. For porous materials like APS TBCs the effect of refraction is smaller [which means a smaller 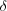 and therefore a smaller 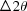 based on equation (10)]. It can therefore be seen that the refraction effect for our measurements can be neglected.

The measured and integrated diffraction patterns were then Rietveld refined by the *TOPAS* program (Bruker AXS, Karlsruhe, Germany). The zero error and Lorentz–polorization factors were fixed to zero. The background was modelled by Chebychev polynomials of second order. The peak shape was a good fit to the Thompson *et al.* (1987 ▸) modified pseudo-Voigt function. The Bondars structure model based on a tetragonal cell (*P*42/*nmc*) was used (Bondars *et al.*, 1995 ▸). The only refinable parameters in the model were the average crystal size and lattice parameters. The 2θ angle range refined was from 6.2 to 10.2°. This relatively simple model gave very reproducible results. The residual stress at each depth was then calculated according to the method introduced above.

After measurement, the samples were ground and polished for microstructure observation on a scanning electron microscope (QUANTA 650, FEI) equipped with an energy-dispersive X-ray spectrometer.

## 4. Results and discussion   

**Figure 2:**
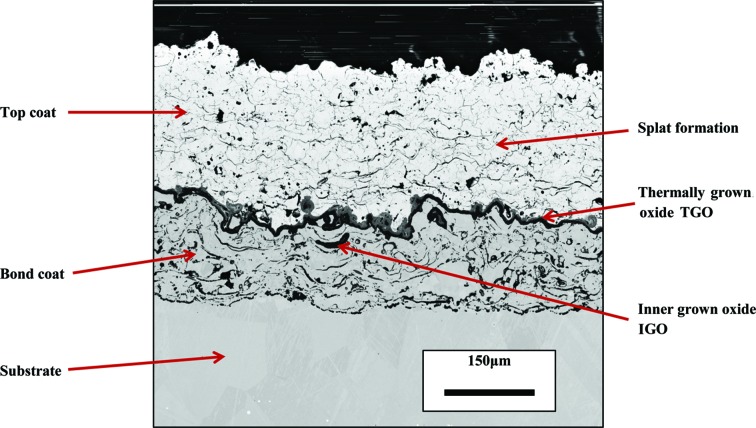
SEM image showing an APS TBC heat treated at 1423 K for 150 h with a ‘splat’ microstructure. Micro cracks between splats are visible, as are the TGO, IGO and interface regions

Fig. 2 ▸ shows the microstructure of the coating system after heat treatment. It can be seen that the coating has flattened pancake ‘splat’ features and the coating is still well bonded to the bond coat. Small and uniform inter-splat cracks can be observed, but no large cracks which could possibly lead to the failure of the TBC. Thus, the residual stress distribution should not be influenced by large cracks. Two layers of TGO can be observed: the top layer which shows a brighter contrast is the spinel and the darker layer at the bottom is alumina (Clarke & Levi, 2003 ▸).

**Figure 3:**
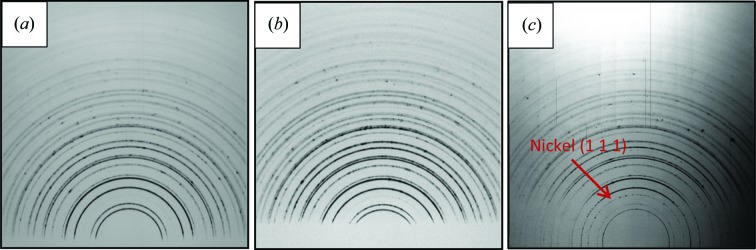
Three diffraction patterns collected from different penetration depths: (*a*) the pattern collected when the beam has grazing incidence on the sample surface; (*b*) the pattern collected when the incident angle is 1.9° (part of the Debye–Scherrer ring has been blocked by the sample) and (*c*) the pattern collected when beam reached the interface; the (111) peak of nickel can be observed

**Figure 4:**
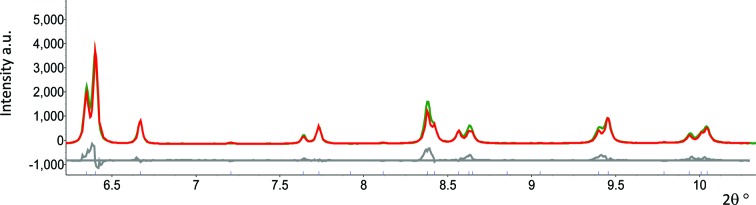
An example of the output from a Rietveld refinement of a diffraction pattern with an incident angle of 1.9°. The observed data are shown in green, the calculated pattern in red, and the difference between the observed and calculated patterns in grey. The ticks show the expected positions of the Bragg peaks. The fit is sufficiently good to measure lattice parameters to the fourth decimal place.

The Debye–Scherrer rings measured with different incident angles are shown in Fig. 3 ▸. It can be seen that half of the Debye–Scherrer ring can be observed on the detector when the beam is at grazing incidence on the sample surface (Fig. 3 ▸ *a*). As the penetration depth becomes larger, the proportion of the Debye–Scherrer ring collected on the detector decreases (Fig. 3 ▸ *b*). When the beam reaches the interface, the peaks of nickel can be observed (Fig. 3 ▸ *c*). This is because the increasing incident angle allows a greater penetration depth. It is noted that there are some small spots on some of the patterns. This is because the beam was adjusted to a very small size (20 × 20 µm). When the beam encounters a crystal which is comparable to the beam size and happens to be in the diffraction condition, the beam will be diffracted like a single-crystal diffraction and result in a relatively bright spot. The XRD pattern after caking and integration is shown in Fig. 4 ▸. It can be seen that most of the peaks are symmetric, which suggests that the spotty pattern does not have a large effect on the integration of the XRD pattern. From the Rietveld refinement, it can be seen that the content of the top coat after heat treatment remains in a single tetragonal prime phase and no trace of phase transformation is observed.

**Figure 5:**
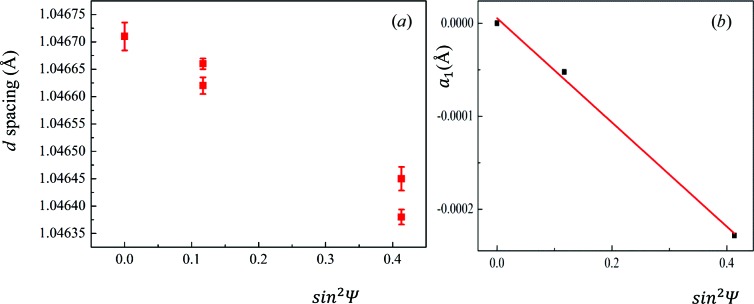
(*a*) A plot of the *d* spacing of the (024) plane against 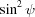. The plot shows a 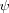 splitting indicating that 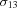 or 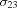 might be nonzero inside the coating. (*b*) A plot of 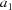 against 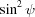 which shows a good linear fit.

Fig. 4 ▸ shows an example of the Rietveld refinement of integrated one-dimensional XRD patterns. It can be seen that the model gives an acceptable weighted profile fit (*R*_wp_ = 7.89%). Since the TBC is mainly composed of a tetragonal prime phase most of the diffraction peaks are ‘doublet’ peaks because of symmetry. The ‘doublet’ peak makes it difficult to get the position of one singular peak accurately. Thus in this measurement, one pair of ‘doublet’ peaks – peaks 024 and 312 – were chosen for the stress analysis. Fig. 5 ▸(*a*) shows an example of the plot of *d* spacing of 024 against 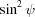. It can be seen that the plot is indicating ‘ψ splitting’, which suggests that 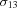 or 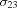 is not zero inside the coating. To calculate the in-plane stress distributed in the coating, the following equation is used in accordance with the work of Noyan & Cohen (2013 ▸):
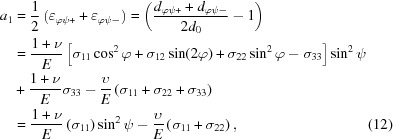


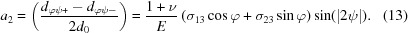
Here 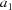 and 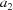 are defined parameters and 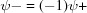. Thus the slope of 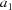 against 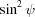 can be given by
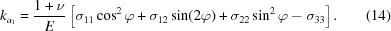



**Figure 6:**
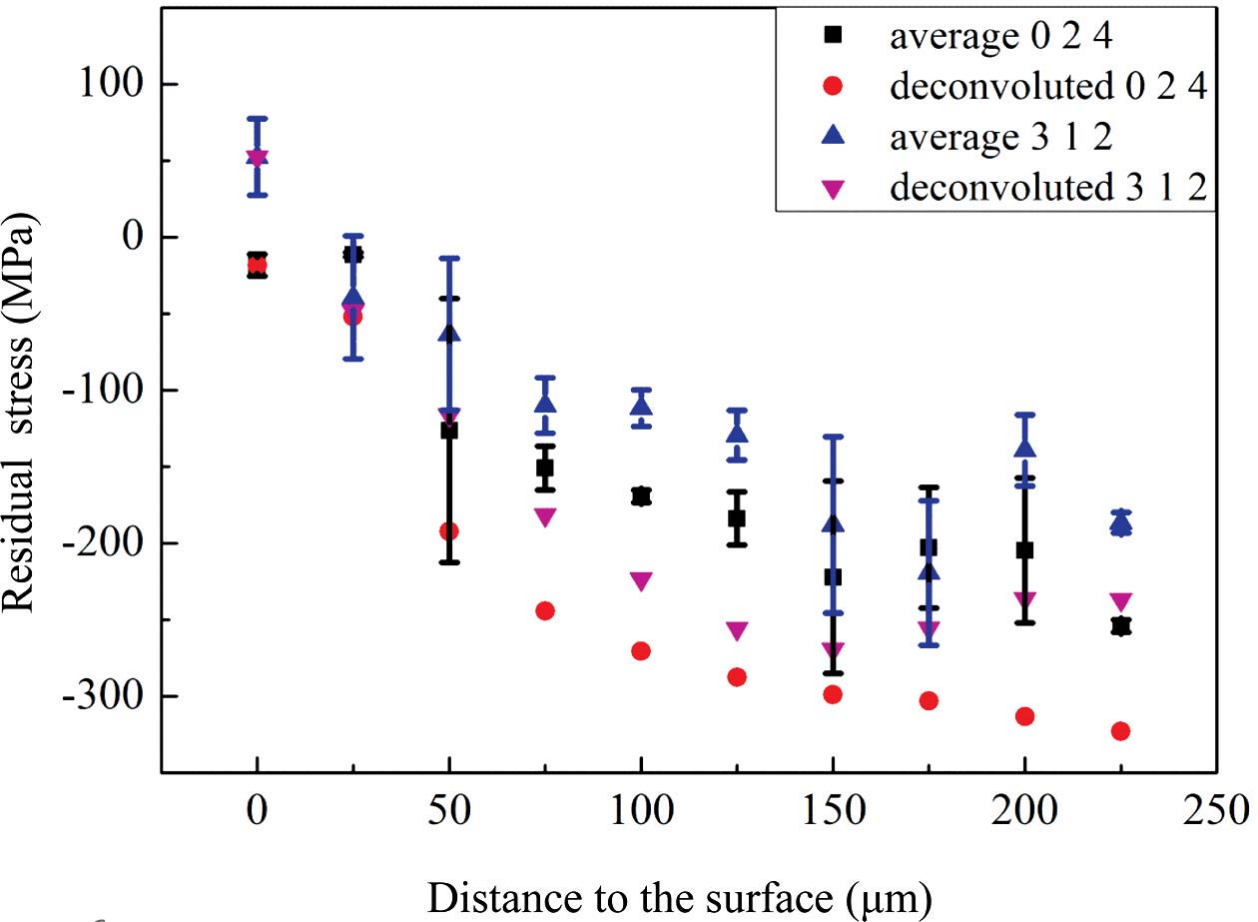
The measured average residual stress and deconvoluted residual stress distribution in the TBC as a function of depth and stress calculated by our analytical model. The measured average stress is compressive and shows a nonlinear trend, increasing from the surface to the interface. The deconvoluted residual stress shows a similar trend to the average stress and the deconvoluted value is larger than the average value. The analytical model shows a linear trend with little difference from the surface to the interface. The deconvoluted value is consistent with the model at the interface but differs at the surface.

**Figure 7:**
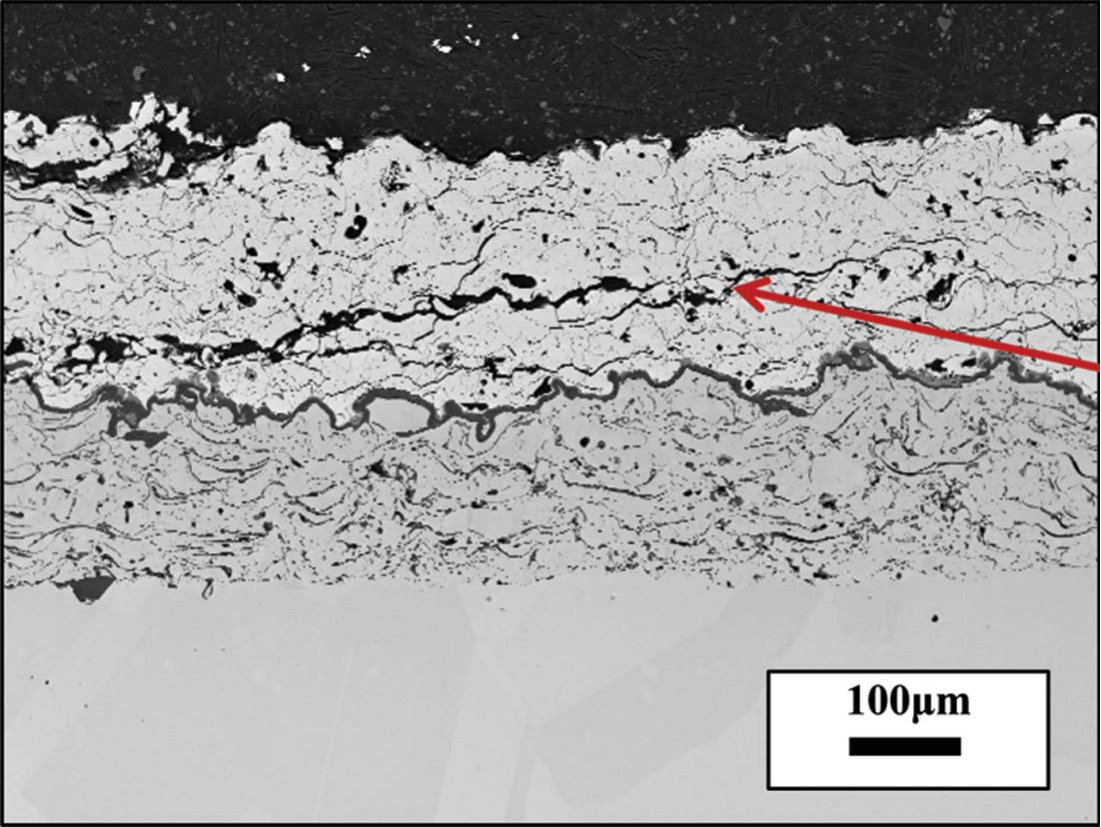
Microstructure of another sample heated treated at 1423 K for 90 h, which shows a large crack (red arrow) deviating from the interface to about the middle of the coating.

In TBCs, the residual stress states can be regarded as in-plane stress states. And in our experiment, no variation in 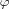 angle was carried out. Thus the equation can be reduced to 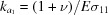, from which the average in-plane stress at different penetration depths can be obtained. Fig. 5 ▸(*b*) gives an example of the fitting, from which it can be seen that the fitting is relatively good. The average residual stress distribution in the TBC as a function of depth using both peaks is shown in Fig. 6 ▸. It can be seen that the results from the two peaks are very similar. For the stress calculated by the 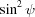 method, the error bars represent the standard deviation on the slope of the linear fitting between 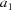 and 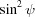, which was achieved by using the *Origin* software (OriginLab Corporation, Northampton, MA, USA) and a linear fitting tool. The average residual stress is compressive near the interface and is generally observed to increase from the surface to the interface with a nonlinear trend. The compressive stress initially increases from 20 to −200 Mpa in the first 125 µm, decreases a little and then increases down to the top coat/bond coat interface. The small difference observed between the stress values from the 024 and 312 reflections can be attributed to the error in calculating X-ray elastic constants. Previous research also gives similar values for the average residual stress. The accurate residual stress value at each depth was calculated by dividing the thermal barrier coating into ten sub-layers (each layer ∼25 µm thick) during the calculation. From Fig. 6 ▸ it can be seen that the deconvoluted residual stress shows a similar trend to the average value. The stress value was observed to increase at first from 20 MPa on the surface to −300 MPa in the middle of the coating. In some samples a fluctuation in the stress gradient was seen (albeit of low statistical significance). In these samples the stress appeared to decrease before increasing again towards the interface. The residual stress in the TBC is generated from the thermal expansion coefficient mismatch between the top coat (11 e^−6^ K^−1^) and the substrate (14 e^−6^ K^−1^). Since the thermal expansion coefficient of the TBC is smaller than that of the substrate a compressive stress is expected in the TBC. The stress can remain in the TBC after cooling owing to the constraint of the substrate. With increasing depth, the constraint from the substrate also increases. Thus, the residual stress increases from the surface to the interface. It can be seen that the deconvoluted residual stress values are slightly larger than the measured average stress values. This is due to the increasing trend of compressive stress from the surface to the interface. However, the trends of the deconvoluted stress values and the average values are quite similar, indicating that the deconvolution carried out was reliable. Previous simulation work shows that the residual stress at the interface is compressive. Luo & Tao (1996 ▸), Zhu *et al.* (2012 ▸) and Zhao & Xiao (2006 ▸) have measured the residual stress by the indentation method and also achieved a value of around −300 MPa at the interface region. We have previously measured the residual strain distribution (Li *et al.*, 2016 ▸) as a function of depth in transmission geometry and found a similar trend. It is noticed that there was a discontinuity of this trend at about 100 µm away from the interface, which can be attributed to the rumpling of the interface and the complex microstructure inside the coating. Fig. 7 ▸ shows the microstructure of a sample heat treated at 1423 K for 90 h. It can be seen that a large crack is generated inside the coating and the crack deviates from the interface towards about the middle of the coating. Cracks in APS TBCs mainly occur at the interface region; however, the propagation of these cracks could interfere with the rumpled interface and generate crack growth towards the middle of the coating. Furthermore, the peak value of the residual stress in the middle of the coating could become the driving force for the large crack to continue to grow. Another possibility could be that, since the microstructure of the TBC is very complex (Fig. 2 ▸) and small cracks can be observed all over the coating, these small cracks could grow larger, leading to TBC failure. The peak value of residual stress situated in the middle of the coating could also act as one of the driving forces for the small defects or cracks to propagate.

## 5. Conclusion   

The residual stress distribution in a TBC as a function of depth has been successfully measured by synchrotron XRD in reflection geometry. The average residual stress is compressive, increasing from the surface (where the value is about 20 MPa) to the interface (about −200 MPa) with a nonlinear trend. The accurate residual stress was deconvoluted by numerical methods. The value increases from 20 MPa on the surface to −300 MPa in the middle of the coating. A fluctuation is indicated in the trend, which shows that the stress decreases and then increases again to the interface. In most reports the stress increases almost linearly towards the interface. In this case, the failure should happen at the interface, which does not explain the failure we observe away from the interface (the crack in Fig. 7 ▸). From our results there is a peak value of the residual stress about 100 µm away from the interface which correlates well with the failure site. One reason for this could be the rumpling morphology: the propagation of the cracks may be stopped by the peak of the rumpling. In this case, the crack will propagate away from the interface. TBC failures can be related to many factors including the type of bond coat. We have indicated how the residual stress distribution and the crack propagation might be related, although this cannot be regarded as definitive without further trials.

Our nondestructive reflection-geometry stress measurement method can also be used to measure the stress in other structures such as brazed joints and ceramic coatings. We further conclude that this reflection deconvolution method may be useful for industrial applications in process monitoring of fully fabricated components. The measured distribution of residual stress can give an indication regarding likely failure modes within whole fabricated components with a sufficiently large statistical sample.
